# The Increasing Challenge of Multidrug-Resistant Gram-Negative Bacilli: Results of a 5-Year Active Surveillance Program in a Neonatal Intensive Care Unit

**DOI:** 10.1097/MD.0000000000003016

**Published:** 2016-03-11

**Authors:** Mario Giuffrè, Daniela M. Geraci, Celestino Bonura, Laura Saporito, Giorgio Graziano, Vincenzo Insinga, Aurora Aleo, Davide Vecchio, Caterina Mammina

**Affiliations:** From the Department of Sciences for Health Promotion and Mother–Child Care “G. D’Alessandro,” University of Palermo, Palermo, Italy; the Azienda Ospedaliera-Universitaria Policlinico “Paolo Giaccone” (MG, CB, VI, CM), Palermo, Italy; Department of Sciences for Health Promotion and Mother–Child Care “G. D’Alessandro” (DMG, AA), University of Palermo, Palermo, Italy; Post-Graduate Residency School in Hygiene and Preventive Medicine (LS, GG), University of Palermo, Palermo, Italy; Post-Graduate Residency School in Pediatrics (DV), University of Palermo, Palermo, Italy.

## Abstract

Colonization and infection by multidrug-resistant gram-negative bacilli (MDR GNB) in neonatal intensive care units (NICUs) are increasingly reported.

We conducted a 5-year prospective cohort surveillance study in a tertiary NICU of the hospital “Paolo Giaccone,” Palermo, Italy. Our objectives were to describe incidence and trends of MDR GNB colonization and the characteristics of the most prevalent organisms and to identify the risk factors for colonization. Demographic, clinical, and microbiological data were prospectively collected. Active surveillance cultures (ASCs) were obtained weekly. Clusters of colonization by extended spectrum β-lactamase (ESBL) producing *Escherichia coli* and *Klebsiella pneumoniae* were analyzed by conventional and molecular epidemiological tools.

During the study period, 1152 infants were enrolled in the study. Prevalences of colonization by MDR GNB, ESBL-producing GNB and multiple species/genera averaged, respectively, 28.8%, 11.7%, and 3.7%. Prevalence and incidence density of colonization by MDR GNB and ESBL-producing GNB showed an upward trend through the surveillance period. Rates of ESBL-producing *E coli* and *K pneumoniae* colonization showed wide fluctuations peaking over the last 2 years. The only independent variables associated with colonization by MDR GNB and ESBL-producing organisms and multiple colonization were, respectively, the days of NICU stay (odds ratio [OR] 1.041), the days of exposure to ampicillin–sulbactam (OR 1.040), and the days of formula feeding (OR 1.031). Most clusters of *E coli* and *K pneumoniae* colonization were associated with different lineages. Ten out of 12 clusters had an outborn infant as their index case.

Our study confirms that MDR GNB are an increasing challenge to NICUs. The universal once-a-week approach allowed us to understand the epidemiology of MDR GNB, to timely detect new clones and institute contact precautions, and to assess risk factors. Collection of these data can be an important tool to optimize antimicrobials use and control the emergence and dissemination of resistances in NICU.

## INTRODUCTION

Multidrug-resistant (MDR) gram-negative bacilli (GNB) are unanimously acknowledged as posing one of the most worrisome challenges in the field of both healthcare associated and community-acquired infections.^1^ Their clinical impact is even more concerning in the neonatal and pediatric care settings where options for treatment are intrinsically limited.^2^ Neonatal intensive care units (NICUs), in particular, can be especially prone to the spread of MDR GNB because of the largely unavoidable exposure to multiple risk factors by critically susceptible subjects.^2,3^ Outbreaks can bring severe consequences in terms of both morbidity and mortality and strict risk mitigation strategies, such as restriction of admissions, ward closure, and, ultimately, disruption of healthcare services.^4,5^ However, endemic circulation of MDR GNB in NICU can be difficult to control as well.^6,7^ Trading of MDR GNB and their resistance determinants between healthcare facilities and community is adding further complexity to this puzzling landscape.^8,9^

Many risk factors and underlying conditions have been identified in association with MDR GNB acquisition in NICU, such as low gestational age, length of stay, use of antibiotics, and exposure to invasive devices.^3,10–13^ Intestinal MDR GNB colonization also plays a role as a risk factor for infection: according with literature data, indeed, up to 50% of infants colonized by MDR GNB, in particular by extended spectrum β-lactamase (ESBL)-producing bacteria, can develop bloodstream infection.^14,15^

In the light of the rapidly evolving epidemiological landscape in reference to logistic and organizational aspects in NICU, characteristics of admitted infants, and pattern of the prominent MDR organisms, the objectives of this study were to describe through a 5-year period of surveillance incidence and trends of MDR GNB colonization and the main characteristics of the most frequently detected colonizing organisms and to identify the risk factors associated with colonization.

## METHODS

### Study Design, Study Setting, and Patients

We conducted a 5-year prospective cohort surveillance study integrated with a program of active surveillance cultures (ASCs). The setting was the tertiary NICU of the teaching hospital “Paolo Giaccone,” Palermo, Italy. This NICU annually admits ∼250 infants of all gestational ages. Because it is associated with the regional reference centre for genetic diseases, the NICU has a high prevalence of neonates with malformation (∼20%), as well as of outborn admissions (∼35%). Moreover, a further 20% proportion of patients is affected by complex conditions requiring subspecialty medical or surgical care. The NICU includes 1 intensive care room consisting of 8 cot spaces and 1 intermediate care room including 8 further cot spaces. The average nurse to patient ratio is 1:3 and 1:4 in the 2 sections, respectively. The NICU ward is open to parents for 2 hours in the morning and 4 hours in the afternoon so that they can be progressively trained in the general care of their child under the guidance and supervision of the staff. Early breastfeeding is encouraged.

Inclusion criteria were admission to NICU between June 16, 2009, and June 15, 2014, a hospital stay of at least 48 hours, and the collection of at least 1 rectal swab. Demographic, clinical, and microbiological data were prospectively collected. At admission demographic characteristics, gestational age, birth weight, inborn or outborn condition, delivery type, APGAR score, and comorbid conditions were recorded. During the NICU stay, qualitative and quantitative data were collected about the following variables: presence of central vascular access devices, endotracheal intubation and mechanical ventilation, nasal continuous positive airway pressure (nCPAP), peripheral catheters, type of feeding (i.e., parenteral nutrition, enteral nutrition with oral suction or gavage, breast milk, formula), surgery, antibacterial drug therapy, length of stay, and survival status at discharge.

Infants were categorized as colonized by MDR GNB when at least 1 rectal swab tested positive.

Clustering of colonization cases by MDR *Escherichia coli* (*E coli*) and *Klebsiella pneumoniae* (*K pneumoniae*) was first hypothesized on the basis of the antibacterial drug resistance profile (both qualitative, i.e., susceptible, intermediately resistant or resistant, and quantitative, i.e., mm of the inhibition halos) and the staying interval in the NICU. Cases were defined as putatively clustered when they were colonized by isolates of *E coli* or *K pneumoniae* with indistinguishable resistance profiles and hospitalized during periods overlapping for at least 48 hours. Clustering was confirmed by pulsed field gel electrophoresis (PFGE) including only indistinguishable and closely related isolates.^16^

Criteria from the Centers for Disease Control and Prevention were applied to define neonatal infection and sepsis.^17^ Both culture proven and culture negative infection cases were counted. Ampicillin–sulbactam and gentamicin were the recommended first-line empiric therapy in most cases of suspected infection, both early and late onset, according to our data on the prevailing bacterial flora in our setting. Usage of third-generation cephalosporins was not recommended.

The study protocol was approved by the Ethics Committee of the Azienda Ospedaliero-Universitaria Policlinico “P. Giaccone,” Palermo, Italy, and informed consent was sought in accordance with the principles of the Declaration of Helsinki. Written informed consent was obtained from the parents or guardians of the neonates.

### Infection Control Interventions

Since June 2009, a surveillance protocol is routinely running, which includes nasal and rectal swabs obtained on a weekly basis from all infants staying in the NICU to monitor the prevalence of carriage with methicillin-resistant *Staphylococcus aureus* (MRSA), MDR GNB and glycopeptide-resistant enterococci. Universal screening at admission is not performed. A policy of appropriate managing of invasive devices is well established in the NICU, including removal of umbilical venous catheter at 72 hours and replacement of any further central venous line within a maximum of 21 days and in any case of suspected/documented bloodstream infection.

Measures taken to control MDR organisms in the NICU include contact precautions, use of dedicated equipment and cyclic training sessions on hand hygiene, and cleaning and disinfection procedures. Moreover, special attention is paid to prevent overcrowding and relative understaffing, to minimize length of hospital stay, and to promote a safe use of invasive devices. Cohorting is adopted, when necessary, but without dedicated nursing staff due to its chronic shortage.

Intensified NICU cleaning is performed when clusters of colonization cases by MDR GNB are suspected. Routine environmental cultures are not performed.

### Microbiological Study

Rectal swabs were collected weekly each Monday. They were pre-enriched in Brain Hearth Infusion broth overnight before being plated on MacConkey agar. Four antibiotic disks, containing gentamicin (10 μg), amoxicillin-clavulanic acid (20–10 μg), meropenem (10 μg), and ceftazidime (30 μg) were placed on each plate before incubation. These antimicrobial drugs were selected in order to include the 2 more frequently used antibacterial drugs in NICU, gentamicin and amoxicillin-clavulanic acid, and 2 further antibiotics, such as ceftazidime and meropenem, that could allow for timely detecting most gram-negative organisms resistant to cephalosporins and/or carbapenems. Colonies growing into antibiotic inhibition halos were subcultured, biochemically identified by home-made biochemical tests and/or API20E strips (Biomérieux, Marne-La Coquette, France), and submitted to antibiotic susceptibility testing. Isolates attributed with *Enterobacter* spp. were not further speciated.

Antibiotic susceptibility testing and ESBL detection were first performed by disk diffusion and double disk synergy test and then confirmed by Etest (Biomérieux, Marcy l’Étoile, France) methods according with the European Committee on Antimicrobial Susceptibility Testing (EUCAST) guidelines.^18^ An isolate was defined as resistant to third-generation cephalosporins, when the inhibition zone diameter of ceftriaxone (30 μg) and cefotaxime (5 μg) was <20 mm and 17 mm, respectively. An isolate was defined as MDR when it was proved to be resistant to at least 3 of the classes of antibacterial drugs under testing (amoxicillin clavulanic, third/fourth-generation cephalosporins, monobactams, aminoglycosides, carbapenems).

*E coli* ATCC 25922 was used as quality control strain.

*E coli* and *K pneumoniae* isolates belonging to each of the epidemic clusters were further characterized by molecular typing. Multiplex polymerase chian reactions (PCRs) were used to amplify genes encoding ESBLs and amplicons were sequenced.^19^*E coli* phylogenetic group was determined by a triplex polymerase chain reaction (PCR) assay as described previously.^20^ Pulsed field gel electrophoresis (PFGE) was performed and electrophoretic profiles interpreted according to standard procedures.^16^*E coli* representative isolates were further characterized by MultiLocus Sequence Typing (MLST) according to the University College Cork (Cork, Ireland) scheme for *E coli* (http://mlst.ucc.ie/mlst/dbs/Ecoli). MLST of selected MDR *K pneumoniae* isolates was also performed according to the guidelines available on the Pasteur Institute MLST website (http://www.pasteur.fr/recherche/genopole/PF8/mlst/Kpneumoniae.html).

### Statistical Analysis

The association between potential risk factors and MDR GNB colonization was analyzed for variables present at admission and during the NICU stay. Time-dependent variables, that is, length of stay, nutrition, invasive procedures, and antimicrobial use, were also measured as days between admission and discharge, death or referral to other healthcare facilities, because patients were assumed to be at risk of further colonization by a different species or strain of MDR GNB after their first isolation.

Categorical variables were compared by using the χ2 test or Fisher's exact test. Continuous variables were compared by the Mann–Whitney *U* test or the ANOVA 1-way test, depending on the distributions. The χ2 for trend was calculated with the Mantel–Haenzsel test. All tests were 2-sided, with *P* < 0.05 considered to indicate significance.

Multivariate logistic regression using the forward step method was used to predict colonization by MDR GNB, ESBL-producing GNB, and multiple MDR GNB. Multivariate logistic regression analysis included all statistically significant variables with *P* < 0.05 in univariate analysis, and gender and gestational age, whether or not they were statistically significant. Goodness-of-fit statistics were calculated and variables were checked for collinearity using conditional indices. The analysis was performed using the softwares EpiInfo, ver. 7, (Centers for Disease Control and Prevention, Atlanta, GA) and SPSS, version 20.0 (IBM SPSS Statistics, IBM Corporation, Armonk, NY).

## RESULTS

### Characteristics at Admission

During the study period, 1152 out of 1374 infants admitted to the NICU fulfilled the inclusion criteria. Among them, 873 (75.7%) were admitted within 24 hours after birth. In >60% of the infants the gestational age was >36 weeks and the birth weight >2500 g. Proportion of inborn was ∼60%.

The annual outborn proportion of admissions raised from 34.9% to 45.7%, even though the increase was not statistically significant (χ2 for trend *P* = 0.16). Proportion of malformation was 19.1%, ranging from a minimum of 17.2% in the 2nd year to a maximum of 20.0% in the 5th year (χ2 for trend *P* = 0.79). Meanwhile, birth weight proved to range from a mean of 2668.5 g (median 2750.0 g, interquartile range [IQR] 2170.0–3230.0 g) in the 1st year to a mean of 2800.9 g (median 2750.0 g, IQR 2280.0 −3290.0 g) in the 5th year, with a peak in the 4th year (mean 2941.9 g [median 2940.0 g, IQR 2250.0 −3320.0 g] (ANOVA, F 3.16; Bonferroni test: 3rd vs 4th year, *P* = 0.007).

The average length of stay was 18.9 days (median 11 days, IQR 7–22 days). Over the study period the median length increased from 9 days, IQR 7–22 days, in the 1st year to 13 days, IQR 8–26 days, in the 3rd year, and again decreased to 11 days, IQR 9–18 days in the 5th year (ANOVA, F 1.71; Bonferroni test: 3rd vs 5th year, *P* = 0.29).

The characteristics of the infants at admission are summarized in Table 1.

**TABLE 1:**
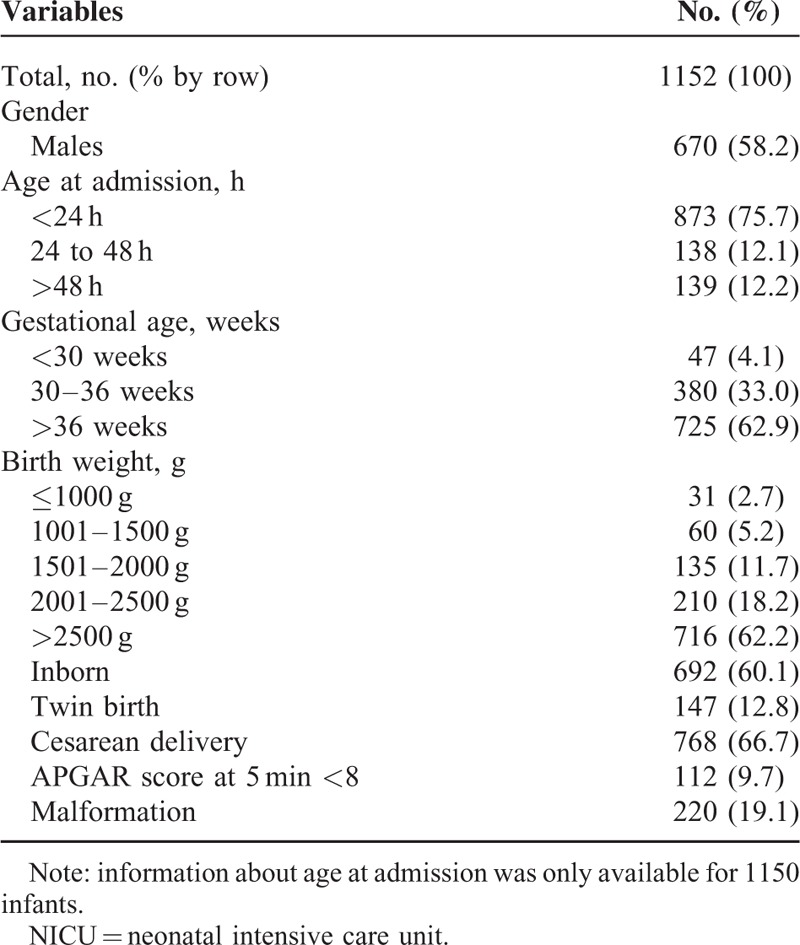
General Characteristics of Patients at Admission to the NICU (June 2009 to June 2014, Palermo, Italy)

### MDR GNB Colonization and Trend in the 5-Year Period

During the 5-year period of surveillance 332 (28.8%) infants tested positive for intestinal colonization by at least 1 species/genus of MDR GNB. The prevalence of colonization showed a steady upward trend from 20.6% in the 1st year to 35.9% in the last year (2nd year 25.0%, 3rd year 32.7%, 4th year 31.3%). Prevalence of colonization by ESBL-producing GNB averaged 11.7% (135 infants out of 1152) ranging between a minimum of 6.6% in the 1st year and a maximum of 20.1% in the 5th year, and a climbing upward trend (2nd year 7.6%, 3rd year 10.8%, 4th year 14.6%).

The mean prevalence of multiple colonization by different MDR GNB genera/species was 3.6% (42 infants out of 1152) with a more stable trend and annual rates ranging between a minimum of 2.1% in the 4th year and a maximum of 5.1% in the 1st year of surveillance.

**FIGURE 1:**
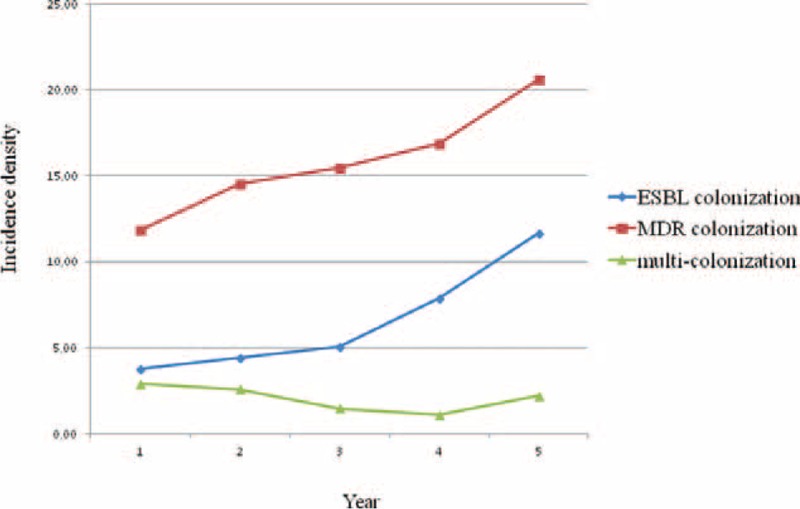
Incidence density per 1000 patient-days of colonization by MDR GNB, ESBL-producing GNB, and multiple MDR GNB through the surveillance period, June 2009 to June 2014, Palermo, Italy. ESBL = extended spectrum β-lactamase, GNB = gram-negative bacilli, MDR = multidrug resistant.

Figure 1 depicts the incidence density of colonization by MDR GNB, ESBL-producing GNB, and multiple MDR GNB through the surveillance period. A significant upward trend was evident for MDR GNB and ESBL-producing GNB with a coefficient of determination of 0.95 and 0.87, respectively. Conversely, incidence of multicolonization did not show significant changes, ranging between a minimum of 1.1 per 1000 patient-days during the 4th year and a maximum of 2.9 per 1000 patient-days during the 1st year.

The proportion of neonates who acquired the most frequently detected genera and species of MDR GNB through the 5-year period was as follows: a mean proportion of 12.9% (range 9.6–14.8%) of neonates acquired *Enterobacter* spp.; 8.1% (range 1.3–17.7%) and 5.4% (range 0–13.4%), respectively, acquired *K pneumoniae* and *E coli*; *Citrobacter* spp. was detected in 3.5% (range 2.4–5.8%) of neonates and *Pseudomonas aeruginosa* in 1.0% (range 0–2.3%). These MDR organisms accounted for ∼95% of the MDR GNB identified from ASCs.

The first ESBL-producing organism to be detected was *K pneumoniae* in 48 infants (35.8%), *E coli* in 31 (23.1), *K pneumoniae* and *E coli* simultaneously in 29 (21.6%), and *Enterobacter* spp. in 20 (14.9%). The median length of stay between admission and the first rectal swab positive was 10 days (IQR 5–18 days). In 90 and 59 infants, respectively, ESBL-producing *K pneumoniae* and *E coli* were detected in 2 to 5 serial rectal swabs.

A range of 1 to 11 MDR GNB positive rectal swabs were obtained from the 42 multi-colonized neonates. Two to 4 different genera/species were identified simultaneously or in the subsequent rectal swabs. *Enterobacter* spp. was identified as one of the colonizing genera in 33 cases. The most frequent association was *Enterobacter* spp. plus *K pneumoniae* (8 neonates) followed by *Enterobacter* spp. plus *Citrobacter* spp. (7 neonates). ESBL-producing *E coli* and/or *K pneumoniae* were identified in 22 cases of multiple colonization.

**FIGURE 2:**
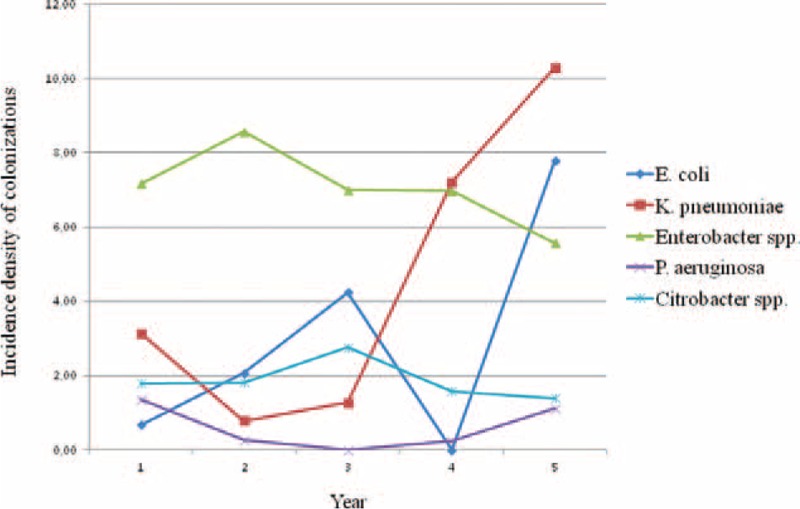
Incidence density per 1000 patient-days of colonizations by *E coli*, *K pneumoniae*, *Enterobacter spp*., *Citrobacter spp*., and *P aeruginosa* through the surveillance period, June 2009 to June 2014, Palermo, Italy.

When looking at the trend of the incidence density during the 5 years (Figure 2), rates of both *E coli* and *K pneumoniae* colonization showed wide fluctuations peaking over the last 2 years. Conversely, incidence densities of colonization by *Enterobacter* spp., *Citrobacter* spp., and *P aeruginosa* remained stable or slightly declining during the study period.

### Risk Factors for MDR GNB Acquisition

Baseline and clinical characteristics of the neonates with MDR GNB and non-MDR GNB are illustrated in Table 2. Most variables present at admission and linked to a condition predisposing to a longer NICU stay, such as twin birth and low birth weight/gestational age, were significantly associated by univariate analysis with being colonized by MDR GNB. Similarly, use of invasive devices, use of antibiotics, and length of stay proved to be significantly associated with MDR GNB colonization. After applying a logistic regression model, the only 2 independent variables proved to be: the APGAR score at 5 minutes ≥8 (odds ratio [OR] 0.757, 95% CI 0.631–0.907), which associated negatively, and the days of NICU stay (OR 1.041, 95% CI 1.003–1.080), which conversely were a significant determinant of colonization.

**TABLE 2:**
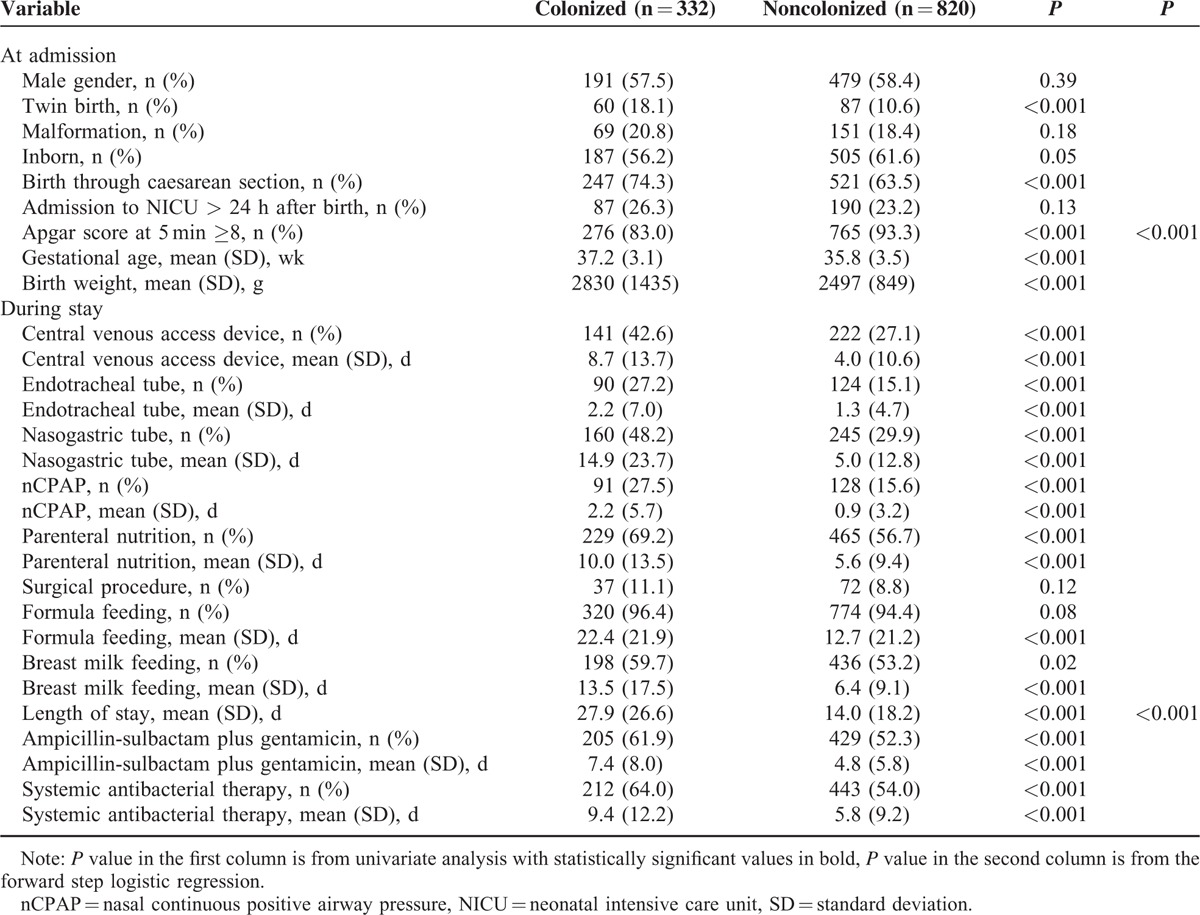
Comparison Between Characteristics at Admission and During NICU Stay of MDR GNB Colonized and Noncolonized Infants, June 2009 to June 2014, Palermo, Italy (n = 1152)

Risk factors for acquisition of ESBL organisms were also analyzed (Table 3). There were statistically significant differences between the 2 groups of colonized and noncolonized neonates regarding the number of days of exposure to central venous access devices, parenteral nutrition, and antibiotic therapy. Parenteral nutrition was also significantly associated with ESBL-producing GNB acquisition as a qualitative variable. After applying the logistic regression model, only the days of exposure to ampicillin–sulbactam were an independent risk factor (OR 1.040, 95% CI 1.009–1.071).

**TABLE 3:**
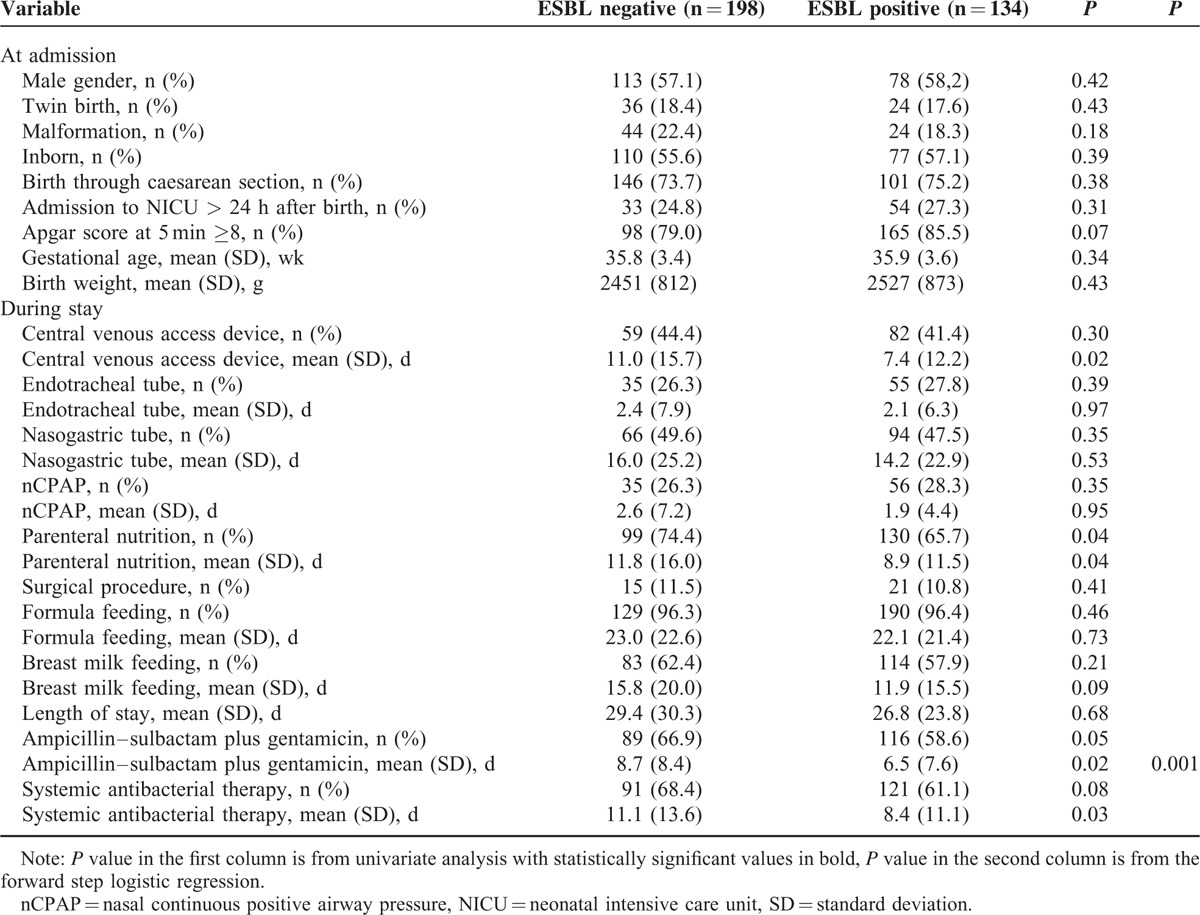
Comparison between characteristics at admission and during NICU stay of ESBL producing bacilli colonized and non colonized infants, June 2009–June 2014, Palermo, Italy (n = 332)

Finally, Table 4 summarizes the results of the analysis of the variables associated with being colonized, simultaneously or subsequently, by multiple genera/species of MDR GNB. Low birth weight/gestational age, exposure to some invasive procedures and antibiotic therapy and days of breast- and formula feeding proved to be significantly associated with multiple colonization. The only independent risk factor by the logistic regression was the number of days of formula feeding (OR 1.031, 95% CI 1.016–1.046). Thirty-three out of 42 (78.6%) multicolonized infants yielded *Enterobacter* spp. versus 146 out of 332 (44.0%) monocolonized (*P* < 0.001). No significant differences were found for other MDR GNB species.

**TABLE 4:**
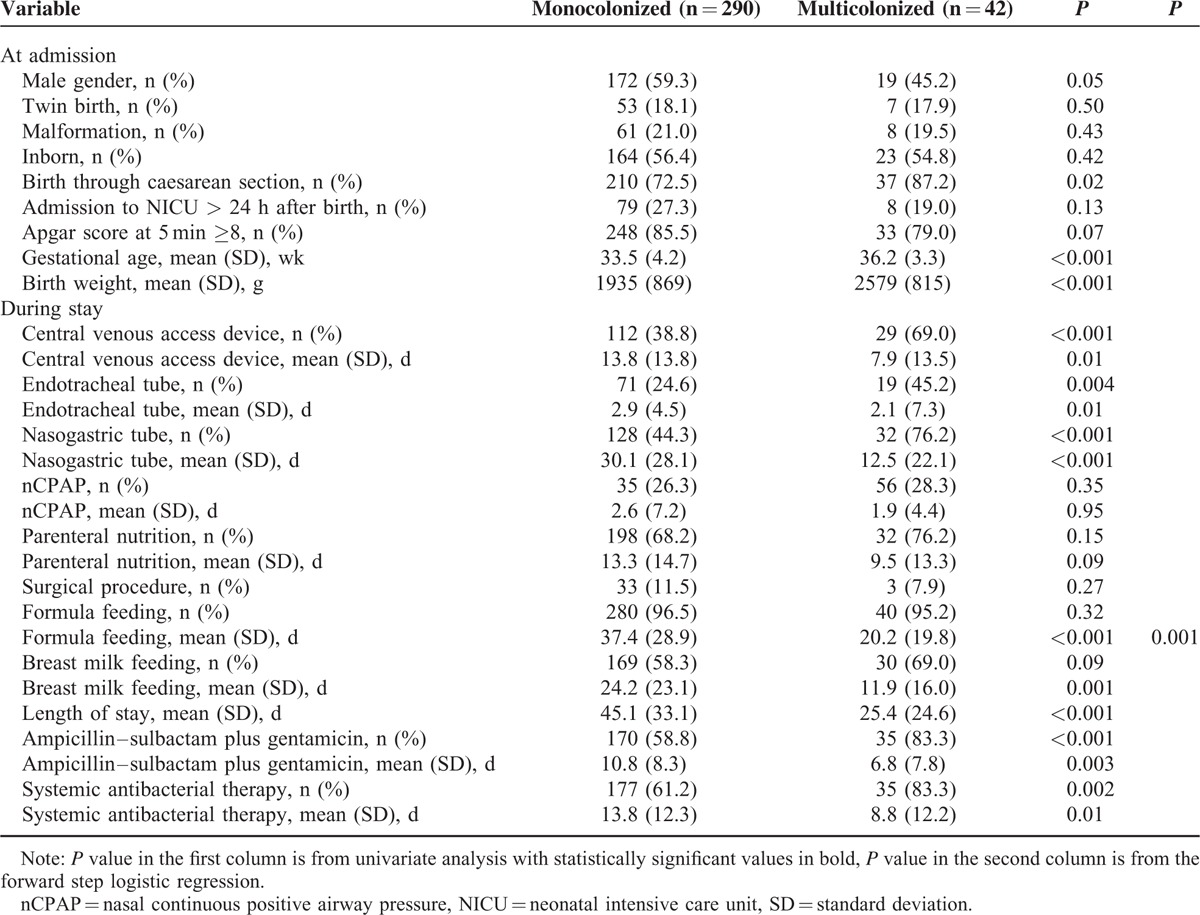
Comparison Between Characteristics at Admission and During NICU Stay of Colonized and Noncolonized Infants by Multiple MDR Organisms, June 2009 to June 2014, Palermo, Italy (n = 332)

Infants with MDR GNB colonization had a significantly higher rate of sepsis than those testing negative (15.5% vs 10.9%, *P* = 0.02). Similarly, a higher rate of sepsis proved to be associated with colonization by multiple MDR GNB species or genera (21.4% vs 11.8%, *P* = .04). Conversely, no significant difference was found between infants colonized by ESBL producing organisms and those who did not (15.1% vs 12.8%, *P* = 0.15).

### Clustering of ESBL-Producing *E Coli* and *K Pneumoniae*

Because of their peculiar epidemiological behavior and the implications of the spread of ESBL-producing *E coli* and *K pneumoniae* isolates in NICU, all isolates were analyzed and screened for putative clustering. Except for 1 patient, who was sequentially colonized by 2 genetically different CTX-M15 and KPC-3-producing *K pneumoniae* isolates, all infants whose serially collected rectal swabs tested repeatedly positive were colonized by genetically indistinguishable isolates.

Table 5 describes the characteristics of the clusters of colonization detected during the 5-year period of surveillance, along with some molecular key features. Some aspects deserve consideration: the great heterogeneity of the causal organisms with the involvement of phylogroups non-B2 and B2 lineages other than ST131 of *E coli* and the detection of 7 different STs of *K pneumoniae* responsible for as many clusters; the predominance of CTX-M-15 as the ESBL genetic determinant in both organisms; the influx of carbapenem-resistant *K pneumoniae* strains with different genetic determinants (KPC-3 and OXA-48) in NICU.

**TABLE 5:**
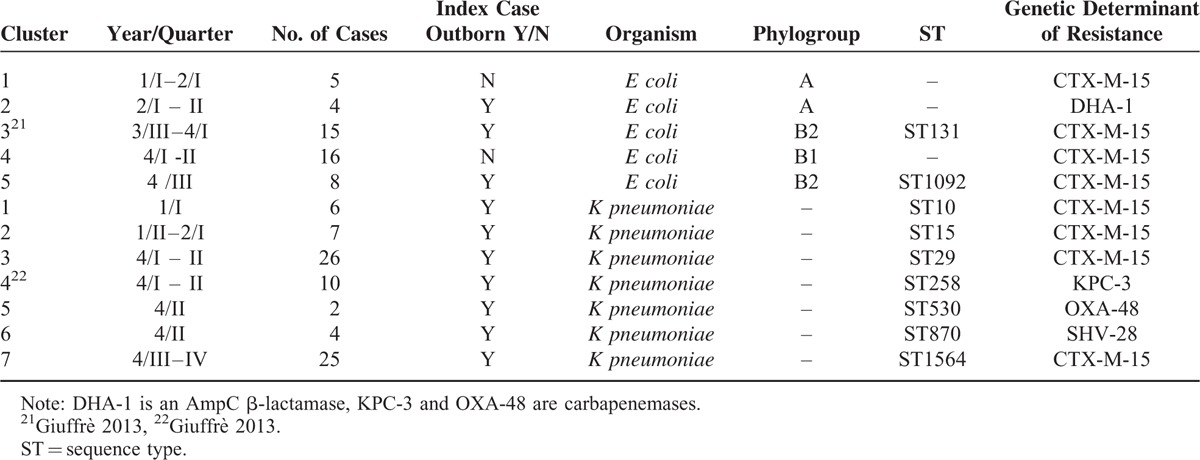
Epidemiological and Microbiological Characteristics of the Clusters of Colonization Cases by ESBL-Producing *Escherichia coli* and *Klebsiella pneumoniae*, June 2009 to June 2014, Palermo, Italy

Finally, it is of interest that in all colonization clusters by *K pneumoniae* and in 3 out of 5 clusters by *E coli* the index case was an outborn infant. Except for 1 patient who had been transferred after 48 hours of stay in the referring NICU, all the remaining infants had been moved to our NICU within 24 hours since their birth time.

## DISCUSSION

In our setting the prevalence of intestinal colonization by MDR GNB was ∼30% and showed an upward trend in the 5-year period under study. This trend was evident when looking at the incidence density and appeared to be largely driven by the spread of ESBL-producing gram negatives. In literature NICU colonization rates as high as 56% of infants colonized by ESBL-producing *Enterobacteriaceae* in Ecuador^23^or 61% by MDR GNB in the Philippines^24^ are reported. However, it is complex to compare data from different NICUs and geographic areas, because of gross heterogeneities in the local epidemiology in each specific area, the structural, logistic, and organizational models of the NICUs and their surveillance policies. A further issue of concern is the different set of antibacterial drugs entering in the definition of an MDR organism as resistant to at least 3 classes of them. In our experience, indeed, only antibacterial drugs of potential use in the treatment of neonatal infections were considered. Conversely, some authors, such as Millar et al,^8^ included within the classes of antibiotics concurring to the identification of an MDR isolate drugs not suitable for neonatal treatment such as tetracycline, colistin, chloramphenicol, and ciprofloxacin. Additionally, samples from infection and colonization cases are frequently merged, occurrence of outbreaks can artifactually inflate the prevalence of a strain/clone, and only some families, such as *Enterobacteriaceae*, can be considered.^8,23^

The primary risk factor identified for MDR–GNB colonization in our study cohort was the number of days spent in NICU. Other previously reported predictors of MDR–GNB colonization, such as low gestational age and /or birth weight, mechanical ventilation, feeding, parenteral nutrition, use of invasive devices, and antibiotic exposure were not identified as independent risk factors.^5,8,10,11,13,23^ The absence of other significant associations strongly suggests the importance of cross-transmission within the NICU.

In our setting, despite the exclusion of cephalosporins from the NICU's antibiotic policy, the average yearly prevalence of ESBL producers colonization was 11.7%, but it cannot be overlooked the upward trend of prevalence and incidence density with roughly doubling figures in the fifth year compared with the first one. It is evident also that CTX-M-15-producing *E coli* and *K pneumoniae* are the main drivers of this trend. Again, comparing prevalences of ESBL-colonized infants in different NICUs and settings is difficult and maybe misleading, because of the known attitude of ESBL-producing organisms to spread by cross-transmission and to trade their ESBL genetic determinants.^23,25^ Conversely, clinical significance of colonization with ESBL-producing strains is well defined. Indeed, in studies from countries with different income distribution and healthcare systems, such as Brazil, Italy, Spain, and Switzerland, between 12% and 50% of neonates colonized with ESBL-producing bacteria developed bloodstream infection.^11,12,26,27^ When comparing within MDR GNB colonized infants those colonized by ESBL-producing and nonproducing organisms, only days of administration of ampicillin–sulbactam proved to be an independent risk factor in the multivariate analysis. Empirical treatment of early onset sepsis by ampicillin–gentamicin is still thought to be appropriate on the assumption of antibacterial drug susceptibility of the community-acquired causative organisms, in particular *E coli*. There is a paucity of neonatal community-acquired ESBL epidemiologic data worldwide, but an increased prevalence of community ESBL-producing gram negatives as etiologic agents of early neonatal sepsis has been reported.^28^ In addition, most ESBL producers demonstrate resistance to aminoglycosides as well.^23,25,29^ The ongoing emergence of ESBL-producing organisms in the community calls for vigilance in monitoring local patterns of antibiotic susceptibility.

It is noteworthy the heterogeneity of the bacterial lineages involved in the clusters with the identification of phylogroups other than B2 and a B2 non-ST131 clone of *E coli* and 7 different STs detected among isolates of *K pneumoniae* involved in as many clusters. Except for the carbapenemase-producing ST258, a well-known pandemic clone, the remaining lineages are not reputed as “high risk” clones and are also genetically unrelated. Two infants were also detected being colonized by an OXA-48 carbapenemase-producing *K pneumoniae* strain, with the index case being born to Tunisian parents. Within the last years, several outbreak and sporadic cases of OXA-48-producing *Enterobacteriaceae* infections have been reported across Europe, most of which related to patients with previous exposure to healthcare facilities in Turkey, Middle East, and northern Africa.^30–32^ Our observations confirm the permeability of NICUs to the import of MDR GNB, in particular ESBL-producing organisms, and suggest that it is possible that as their prevalence is increasing in the community, the likelihood of their introduction into an NICU is increasing as well.

It is also interesting to observe the wide predominance of outborn infants as index cases of the colonization clusters. Most of them had been transferred in the 24 hour-interval after their birth, but it is also well known that in low endemic setting the acquisition of MDR GNB can be very precocious.^33^ However, because screening at admission was not performed, an MDR GNB acquisition in our NICU cannot be ruled out. Recently, epidemiological investigations, as well as mathematical modeling, have shown that the transmission dynamics of multidrug-resistant bacteria crucially depend on the structure of the underlying healthcare network, as shaped by inter-institutional referral pattern of patients.^34,35^ A tertiary NICU, such as that under study, occupies a central position in the neonatal healthcare network and is, consequently, especially vulnerable to the spread of previously acquired pathogens.^36^ Our data suggest to include in the ASC program an admission screening for MDR organisms of all outborn patients, or at least of those at higher risk because referred from other NICUs. The need of a network based, coordinated regional approach to infection prevention has also been recently emphasized by authoritative healthcare agencies.^37^

Finally, the only independent risk factor for colonization by multiple MDR GNB species and/or genera was the number of days of formula feeding. Infant formula is a well-described pathway for healthcare-acquired infections in NICUs.^38,39^ Intrinsic and extrinsic contaminations of powdered infant formula can occur during manufacture, during reconstitution through the use of contaminated water, utensils, work surfaces, at the time of feeding, in particular by enteral tubing with biofilm, or because of inappropriate storage.^40^*Enterobacter* spp. as well as species/genera that were not speciated (e.g., *Pantoea* spp., *Cronobacter* spp., *E hormaechei*, *E vulneris*, etc.) have been repeatedly detected in powdered formula.^41^ This could have likely contributed, along with the deficiency of a commensal flora promoted by breast milk, to the high background prevalence of *Enterobacter* spp. colonization in the NICU.

Our study has some limitations. Because weekly surveillance cultures were scheduled at the same day for all infants staying in NICU regardless of their interval of time since admission, it was not possible to define when the colonization by MDR GNBs was acquired. This could be especially relevant for the outborn infants. However, the need to ensure high compliance to the ASC program was considered prioritary and, consequently, according with some authors choosing a similar option, screening of each neonate at specific and different time was ruled out.^42^ Moreover, microbiological methods of isolation and identification were relatively rough and lacking of accuracy in speciation of MDR GNBs. Our aim was, indeed, to obtain epidemiologically meaningful information quickly, minimizing at the same time the laboratory costs and workload.^43^ The role as risk factors of logistic and organizational features such as overcrowding, understaffing, and infection control practices, for example, hand hygiene, was not assessed. However, the prominent role of the length of stay in NICU as the only independent variable positively associated with MDR GNB acquisition as well as the descriptive characteristics of the epidemic clusters, highlight cross-transmission as the most likely route of colonization. The association between colonization and infection was only described but an accurate assessment of the clinical outcome was beyond the purposes of this study. Scarcity of data from clinical cultures and limitations related to the low detection rate of the neonatal blood cultures contributed to this option. Finally, the case mix of participants in our NICU was strongly influenced by the level of the unit and the available services, mainly surgery. Consequently, generalizability of our findings could be limited.

## CONCLUSIONS

In conclusion, our study confirms an increasing challenge of MDR GNB to NICUs. Due to the intrinsic restrictions of the available treatment options, the upward epidemiological trend of these organisms is a significant threat to patients admitted to NICUs, particularly to high-risk infants.

Though there is no agreement about the most cost-effective strategy of the surveillance cultures in NICU and a universal once-a-week approach is a compromise solution to warrant a high and continued adherence, this intervention allowed us to thoroughly understand the changing epidemiology of MDR GNB in the NICU setting, to timely detect the import and spread of new multiresistant clones and institute contact precautions, and to assess risk factors for MDR GNB colonization. Additionally, collection of these data was an important tool to optimize antimicrobials use and control the emergence and dissemination of resistances. The frequent need to treat in the absence of culture results makes it even more significant the contribution provided by the active surveillance programs.
